# T-Cell Subpopulations αβ and γδ in Cord Blood of Very Preterm Infants: the Influence of Intrauterine Infection

**DOI:** 10.1007/s00005-013-0244-z

**Published:** 2013-08-20

**Authors:** Agata Serwatowska-Bargieł, Maria Wąsik, Maria Katarzyna Kornacka, Elżbieta Górska, Robert Kozarski

**Affiliations:** 1 Neonatal and Neonatal Intensive Care Department, Medical University of Warsaw, Princess Anna Mazowiecka Hospital, Karowa 2, 00-315 Warsaw, Poland; 2 Department of Laboratory Diagnostics and Clinical Immunology of Developmental Age, Medical University of Warsaw, Warsaw, Poland; 3 University of Hertfordshire Health and Human Science Research Institute, CLiCIR, Hertfordshire, UK

**Keywords:** CD3^+^ cells, Gamma/delta T cells, Preterm newborn, Intrauterine infection

## Abstract

Preterm infants are very susceptible to infections. Immune response mechanisms in this group of patients and factors that influence cord blood mononuclear cell populations remain poorly understood and are considered insufficient. However, competent immune functions of the cord blood mononuclear cells are also described. The aim of this work was to evaluate the T-cell population (CD3^+^) with its subpopulations bearing T-cell receptor (TCR) αβ or TCR γδ in the cord blood of preterm infants born before 32 weeks of gestation by mothers with or without an intrauterine infection. Being a pilot study, it also aimed at feasibility check and assessment of an expected effect size. The cord blood samples of 46 infants age were subjected to direct immunofluorescent staining with monoclonal antibodies and then analyzed by flow cytometry. The percentage of CD3^+^ cells in neonates born by mothers with diagnosis of intrauterine infection was significantly lower than in neonates born by mothers without infection (*p* = 0.005; Mann–Whitney *U* test). The number of cells did not differ between groups. Infection present in the mother did not have an influence on the TCR αβ or TCR γδ subpopulations. Our study contributes to a better understanding of preterm infants’ immune mechanisms, and sets the stage for further investigations.

## Introduction

Preterm infants, especially those born before 32nd week of gestation, are at a high risk of developing life-threatening conditions, such as a respiratory distress or sepsis (Maxwell et al. 2006; Stoll et al. 2005). One of the main causes of perinatal morbidity and mortality of these patients is the intrauterine infection of bacterial etiology proven to be responsible for preterm delivery (Goldenberg et al. 2008; Romero et al. 2007; Stoll et al. 2005). Neonates born by mothers with histopathologic diagnosis of chorioamnionitis are more likely to suffer from such long-term complications as periventricular leukomalacia and cerebral palsy (Duggan et al. 2001). Immune response mechanisms in neonates, especially in preterms, are not well established and they are considered very premature and insufficient to fight infections (Marodi 2006; Schelonka and Infante 1998). However, even during the fetal period immune response to mother’s infection is observed (Zhao et al. 2002), and cord blood mononuclear cells are known to secrete significant amounts of proinflammatory cytokines (Cairo et al. 2008; Chipeta et al. 2000; Koehler et al. 2008). Yet the development of the human immune system during the prenatal and perinatal period is not precisely understood. Factors that have an influence on different immune cell populations remain unclear.

In this work, which is a pilot study for further research, the T-cell population and its subpopulations expressing T-cell receptor (TCR) αβ and γδ were studied in very preterm infants, born before 32 weeks gestational age. T cells are a part of acquired immunity and would rather seem to play a minor role in newborn immune responses. However, their subpopulation bearing the γδ TCR is considered as the first line of defense in bacterial infections (Follows et al. 1992; Jouen-Beades et al. 1997; Konigshofer and Chien 2006; Moore et al. 2000; Nakasone et al. 2007; Xiong and Raulet 2007). There are studies on human neonates, in which γδ T cells were found highly reactive to pathogens compared to the subpopulation αβ. Other studies describe higher percentage of memory cells among the γδ subpopulation in cord blood than in other subpopulations of cells (De Rosa et al. 2004; Gibbons et al. 2009). As the preterm birth is frequently related to bacterial intrauterine infection (Goldenberg et al. 2008; Romero et al. 2007; Sherman et al. 1997), the present study was done to evaluate the effect of this kind of infection on T-cell population (CD3^+^ cells, with subsets αβ and γδ) in newborns. We hypothesized, based on the above findings, that the presence of bacterial infection in the mother during delivery would increase the number and/or percentage of γδ T cells in cord blood of preterm newborn.

## Materials and Methods

The study material consisted of 46 newborns born before completing 32 weeks of gestation in Princess Anna Mazowiecka Hospital and hospitalized in the Neonatal and Neonatal Intensive Care Department of the Medical University of Warsaw, during the years 2007–2009. Twenty-nine of the newborns (group I) were children of mothers with intrauterine infection. Within this group 14 newborns did not develop signs of neonatal infection and 15 presented with clinical and laboratory signs of infection. Seventeen neonates (group II) came from mothers without infection. The main characteristics of the groups of studied infants are presented in Table 1. Mothers with any TORCH (toxoplasmosis; other—measles, influenza, syphilis; rubella; cytomegalovirus; herpes)infection diagnosis, and babies with congenital defects were excluded from the study. Signing the informed consent by the parents was a necessary inclusion criterion.

**Table 1:** Main characteristics of the studied groups

	Group I (*n* = 29)	Group II (*n* = 17)	*p* value*	Group Ia (*n* = 14)	Group Ib (*n* = 15)	*p* value*
Gestational age (weeks)						
Median (range)	29 (24–31)	30 (26–31)	0.04	29 (25–31)	28 (24–31)	NS
Birthweight (g)						
Median (range)	1,088 (640–1,650)	1,460 (720–1,860)	0.022	1,305 (640–1,560)	1,020 (660–1,650)	NS
Sex M/F						
Number (%)	15/14 (51/49 %)	8/9 (47/53 %)	NS	6/8 (43/57 %)	9/6 (60/40 %)	NS
Type of delivery V/C						
Number (%)	15/14 (51/49 %)	3/14 (18/82 %)	0.048	5/9 (36/64 %)	10/5 (67/33 %)	NS
Antibiotic treatment of the mother						
Number (%)	24 (83 %)	8 (47 %)	0.027	12 (85 %)	12 (80 %)	NS

*M* male, *F* female, *V* vaginal delivery, *C* caesarian section, *NS* not significant

* Values assess the differences between the fractions

The study was approved by the Ethical Committee of the Medical University of Warsaw.

The maternal infection was initially diagnosed based on the clinical signs and/or laboratory tests (C-reactive protein: CRP, number of white blood cells: WBC). Results of bacterial cultures of maternal vaginal smears and amniotic fluid were acknowledged later, as well as histologic results. In the preterm birth, intrauterine infection may manifest with very subtle clinical signs. Therefore, any positive laboratory findings, together with positive bacterial cultures and/or histologic chorioamnionitis, were sufficient to qualify the patient to the group with infection (Locksmith and Duff 2001; Newton 2005; Romero et al. 2007; Snyder et al. 2007). Intravenous antibiotic therapy is routinely administered to most mothers during preterm labor (Baltimore 2007; Locksmith and Duff 2001; Snyder et al. 2007). Mothers colonized with group B streptococcus received ampicillin, and those colonized with other species received ampicillin plus sulbactam. In the case of allergy to penicillins, cefuroxime or erythromycin was administered. In group I, five mothers were considered untreated. Those were the mothers who delivered their babies either before receiving their first dose or right after that dose (up to 30 min), which was considered rather ineffective. In group II, 8 of 17 mothers received the treatment. Those mothers either had vaginal colonization with pathogenic flora or premature rupture of membranes, which were considered as the infection risk factor.

Diagnosis of neonatal infection was established by repeated blood testing for complete blood count with differential count and CRP. Blood cultures were obtained before administering routine antibiotic therapy (ampicillin and netilmycin). About 48 h after birth, the neonates from group I were assigned to groups Ia (14 neonates) and Ib (15 neonates) according to clinical and laboratory findings. These were the groups where children did not and did develop infection, respectively (Polin and Spitzer 2001).

The cord blood samples (about 1 cm^3^) were collected at birth to the EDTA tubes and subjected to analysis. Number of blood leukocytes was estimated on Coulter LH750 hematological analyzer (Beckman Coulter, Inc., USA) and their concentration was adjusted to 5,000/μl either by dilution with 0.9 % NaCl or centrifugation. Then, the blood samples were stained with the following monoclonal antibodies: anti-CD3 FITC clone: W31, anti-CD3 PE clone: 11F2, anti-TCR α/β FITC clone: WT31, anti-TCR γ/δ PE clone: 11F2 and FITC i PE isotype controls, all purchased from the Diag-Med authorized distributor of BD Biosciences (USA). A direct immunofluorescent staining of the whole blood was performed according to BD Biosciences procedures. The red cells were lysed and binding to monoclonal antibodies was fixed with Uti-Lyse Erythrocyte Lysing Reagents (Dako, USA). This procedure was performed according to the Dako procedures. Routinely, 1 × 10^4^ cells were analyzed by two-color flow cytometry on Cytomix FC 500, Beckman Coulter. The percentage and number of total T cells (CD3^+^ cells), and then of αβ (CD3^+^ TCR αβ) and γδ (CD3^+^ TCR γδ) subpopulations were determined in the population of lymphocytes gated on lymphocyte base FCS/SCC diagram.

Given that the normality assumption was rejected at a baseline 0.05 significance level in majority of the results (Shapiro–Wilk test), the independent group comparisons were performed using a non-parametric Mann–Whitney approach. Due to a small sample size, a continuity correction of the testing statistics was applied. The Bonferroni correction method was used to adjust the baseline significance level for a multiple testing bias. All the analyses and graphics were performed using the software R version 2.15.2 (R Foundation for Statistical Computing, Vienna, Austria).

## Results

Table 1 presents the main features of studied groups of neonates, gestational age, birthweight, type of delivery, and the mother antibiotic therapy type. The median CRP in mothers from group I was 33 mg/l (normal range 0–10 mg/l; 25th–75th percentile 17–67 mg/l), median of WBC was 16.2 × 10e9/l (25th–75th percentile 12.9–18.8) (normal range 4–11 × 10e9/l). The median CRP for group II was 5 mg/l (25th–75th percentile 4–9 mg/l), and median WBC 11.6 (25th–75th percentile 10.1–15). Those values differed significantly between groups (Mann–Whitney *U* test, *p* < 0.001 for CRP, *p* < 0.01 for WBC). Infants of group Ia and II had CRP ≤10 mg/l and WBC within normal range for their age, while infants from group Ib had median of CRP 17 mg/l. In addition, three of them had also positive blood cultures.

**Fig. 1:**
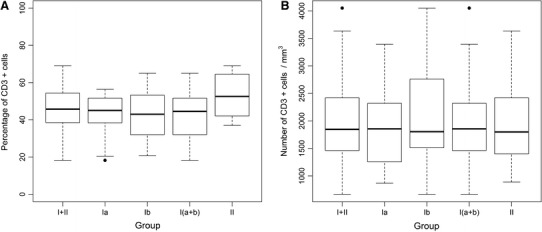
Percentage (**a**) and number (**b**) of CD3^+^ cells in cord blood of studied neonates. Group I—neonates of mothers with intrauterine infection, in this Group Ia—neonates without signs of infection, Group Ib—neonates with diagnosed infection, Group II—neonates of mothers without infection

The total WBC in the studied infants ranged from 3.5 to 45.3 × 10e9/l, and the lymphocytes from 20.6 to 88.8 % of total leukocytes, which gave the number of lymphocytes from 1,796 to 18,648/mm^3^. The numbers and percentages of T lymphocytes (with αβ and γδ T cells) are presented in detail in Table 2. The percentage of CD3^+^ cells was significantly lower in group I than in group II (*p* < 0.01). There was no significant difference in number of CD3^+^ cells between group I and II. No significant differences in CD3^+^ cell number or percentage were found between uninfected and infected newborns of mothers with intrauterine infection (group Ia vs. Ib, *p* not significant) (Fig. 1).

**Table 2:** The percentages and numbers of CD3^+^ and subpopulations CD3^+^ TCR αβ and CD3^+^ TCR γδ in the studied groups of newborns

	Group I Me (25th–75th percentile)	Group II Me (25th–75th percentile)	*p* value	Group Ia Me (25th–75th percentile)	Group Ib Me (25th–75th percentile)	*p* value
CD3^+^ /mm^3^	1,856 (1,488–2,266)	1,838 (1,326–2,427)	NS	1,895 (1,353–2,250)	1,804 (1,525–2,518)	NS
CD3^+^ %^a^	44 (32.4–51.4)	52.6 (42.2–64.2)	0.005	45.3 (36.7–52.1)	43 (20.7–65)	NS
CD3^+^ αβ /mm^3^	1,730 (1,355–2,188)	1,713 (1,071–2,216)	NS	1,818 (968–1,900)	1,699 (1,432–2,308)	NS
CD3^+^ αβ %^b^	92 (87–96)	92 (90–94)	NS	89 (82.5–96.5)	92 (89–95)	NS
CD3^+^ γδ /mm^3^	38 (24–60)	38 (22–52)	NS	33 (23–69)	50 (38–59)	NS
CD3^+^ γδ %^b^	2.1 (1.5–3)	1.9 (1.5–2.5)	NS	1.8 (1.1–3.2)	2.4 (1.9–2.7)	NS

Group I: neonates of mothers with intrauterine infection, in this Group Ia: neonates without signs of infection, Group Ib: neonates with diagnosed infection, Group II: neonates of mothers without infection

*Me* median, *NS* not significant

^a^Of all lymphocytes

^b^Of CD3^+^ lymphocytes

**Fig. 2:**
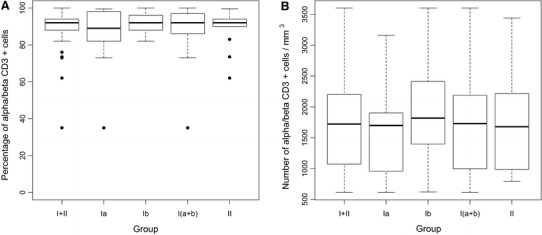
Percentage (**a**) and number (**b**) of CD3^+^ TCR αβ cells in cord blood of studied neonates. Group I—neonates of mothers with intrauterine infection, in this Group Ia—neonates without signs of infection, Group Ib—neonates with diagnosed infection, Group II—neonates of mothers without infection

**Fig. 3:**
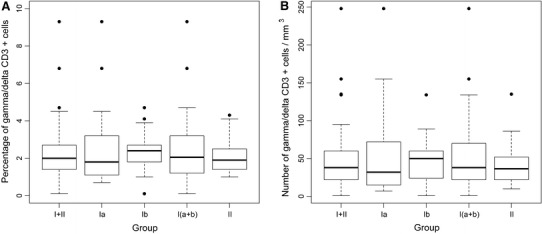
Percentage (**a**) and number (**b**) of CD3^+^ TCR γδ cells in cord blood of studied neonates. Group I—neonates of mothers with intrauterine infection, in this Group Ia—neonates without signs of infection, Group Ib—neonates with diagnosed infection, Group II—neonates of mothers without infection

The percentages and numbers of CD3^+^ TCR αβ and CD3^+^ TCR γδ cells did not differ significantly between groups I and II nor groups Ia and Ib (Figs. 2, 3).

## Discussion

The present study was conducted to evaluate the association between intrauterine infection of bacterial etiology and the T-cell population in newborn’s cord blood. The mechanisms of newborn immune response remain unclear, and this is especially true for very preterm newborns. Some previous studies on neonatal immunology suggest that fetal and early neonatal cytokine production is biased toward Th2 type response. Suppression of interferon (IFN)-γ secretion (the main cytokine in Th1 response) is due to a higher expression and secretion of interleukin 4 by Th2 cells (Langrish et al. 2002; Zaghouani et al. 2009). An increased production of pro-inflammatory cytokines such as IFN-γ may cause damage of the placenta and therefore lead to miscarriage, preterm birth, or serious fetal injury (Marodi 2006; Romero et al. 2007). Therefore, the Th1 response suppression seems to be an important evolutionary mechanism, necessary for proper pregnancy maintenance.

Our results prove that intrauterine infection affects the T-cell population. The percentage of CD3^+^ cells in newborns born by mothers with intrauterine infection was significantly lower than in the newborns of mothers without infection (*p* = 0.005). This outcome is in line with Juretic et al. (2001) findings, who studied lymphocyte subsets in preterm infants too. Other authors, however, did not describe this effect. In Kotiranta-Ainamo et al. (1999), the percentage of CD3^+^ cells in newborns with intrauterine infection was higher than in uninfected newborns, and in Matsuoka et al. (2001) the difference in CD3^+^ lymphocytes between groups of infected and uninfected infants was not significant. These discrepancies are probably due to a small number of patients included in the studies, which is not unusual in this type of research. Most of the cited literature is based on the groups of patients <50 (Juretic et al. 2001; Kotiranta-Ainamo et al. 1999; Matsuoka et al. 2001; Mazur et al. 2004). This probably results from problems with obtaining cord blood sample in the most immature neonates, who, in most cases, require resuscitation at birth, engaging all the present personnel. The other reason for these discrepancies might be different gestational age of the studied populations. The study presented here consisted only of preterm neonates, similarly to the study conducted by Juretic et al. (2001). The two other studies of Kotiranta-Ainamo et al. (1999) and Matsuoka et al. (2001) included also term infants, which might be a matter of significance for the immunologic mechanisms. Because of the limited number of studies and the fact that only term newborns are usually considered, it is quite difficult to discuss the above results. Our study is one of the very few performed on a population of children born before completing 32 weeks of gestation. Another problem is that different authors evaluate different subpopulations of mononuclear cells, therefore, the results are not comparable. Some studies assess the activation of cells measured by cytokine concentrations (Gibbons et al. 2009; Matsuoka et al. 2001), but they do not consider the quantity evaluation of cell populations.

Because the gestational age of the studied groups differed significantly (lower gestational age in the group of neonates born by infected mothers), as well as infants’ birthweight, our outcomes must be interpreted with caution. This fact, although it might raise doubts of statistics credibility, is consistent with the known dependence of intrauterine infection frequency and gestational age (Goldenberg et al. 2008; Maxwell et al. 2006; Romero et al. 2007). Intrauterine infection is more often present in the most immature and therefore the smallest neonates.

The present study considers also the role of a unique subpopulation of T cells: the CD3^+^ TCR γδ. This subset is a subject of many studies, appearing, among other functions, as the first line of defense in bacterial infections (Follows et al. 1992; Jouen-Beades et al. 1997; Konigshofer and Chien 2006; Moore et al. 2000; Nakasone et al. 2007; Xiong and Raulet 2007). The reaction of the γδ T cells to bacterial antigens and their ability to secrete IFN-γ is spontaneous and independent from the major histocompatibility complex. This is a very unique feature among lymphocytes, and this subpopulation is often described as a “bridge” between innate and acquired immunity (Beetz et al. 2008). The γδ T cells are often described as an immature or primitive subpopulation of T cells which develops early during ontogeny (Konigshofer and Chien 2006; Xiong and Raulet 2007), and might be a significant part of preterm infants’ immune response. Therefore, we found it interesting to investigate this subpopulation in preterm infants. Their presence and increased activity in the case of bacterial intrauterine infection might be a partial explanation for the increased Th1 type response in the otherwise Th2-biased immune system of the newborn.

There is a very limited number of studies on this subset of cells in intrauterine infection, while most of them are performed on term infants. Our results show no significant differences in the CD3^+^ TCR γδ cell number or percentage in cord blood of preterm infants of mothers with bacterial intrauterine infection compared to newborns from uninfected mothers. The observation here in preterm infants agree with the data of Mazur et al. (2004) seen in term newborns. Data reported by Gibbons et al. (2009) show an increased IFN-γ production by the γδ subset of CD3^+^ cells in preterm newborns. Unfortunately, these authors did not show any quantity evaluation results of these cell subsets. Taken together, the above results might suggest that the role of the γδ T cells in intrauterine infection is based more on an increased cell activation measured by cytokine production, rather than increased cell number or percentage. However, to prove this, further studies, taking into account both the quantity assessment and cell activation, are required.

In conclusion, the presented results might contribute to a better understanding of the immune response in very preterm infants. It sets the stage for further studies with a greater number of patients to fully assess the mechanisms involved in premature infants’ immunologic reactions. Such a study is planned for the near future.
